# Self-determined use of provided powered oral hygiene devices leads to improved gingival health after 1 year: a longitudinal clinical trial

**DOI:** 10.1186/s12903-024-04313-7

**Published:** 2024-05-14

**Authors:** I. Deeg, M. J. Wicht, A. G. Barbe, S. H. M. Derman

**Affiliations:** Polyclinic for Operative Dentistry and Periodontology, University of Cologne, Faculty of Medicine and University Hospital Cologne, 50931 Cologne, Germany

**Keywords:** Concordance, Gingivitis, Oral hygiene, Microdroplet device, Powered toothbrush

## Abstract

**Purpose:**

Our study aimed to evaluate the long-term concordance and acceptance when using powered devices for everyday oral hygiene routine and gingival health in patients showing papillary bleeding.

**Patients and methods:**

Thirty-one participants were recruited at the dental clinic of the University Hospital of Cologne, Germany, over a 6-week duration. At baseline, a standard dental check-up was performed, including oral hygiene indices and documentation of oral hygiene devices used. The study consisted of two consecutive phases: the first (motivational trial) was designed to prove the effectiveness and safety of a microdroplet device and a powered toothbrush compared to dental floss and a manual toothbrush over a period of 4 weeks. The second (observational) phase began with all participants receiving the powered oral homecare devices. Participants were able to use their oral hygiene measures of choice over an unsupervised period of 1 year. All participants were then rescheduled for a routine dental check-up, where oral hygiene indices and oral hygiene devices used were reevaluated.

**Results:**

After 1 year, 93.3% of participants stated they performed interdental cleaning on a regular basis (baseline 60.0%). The percentage using a powered toothbrush increased from 41.9% (baseline) to 90.0% after 1 year. Oral hygiene parameters had improved after both the motivational trial and observational phases compared to baseline (papillary bleeding index *p* = .000; Rustogi Modified Navy Plaque Index *p* < .05; Quigley-Hein Index *p* = .000).

**Conclusion:**

In the long term, participants preferred using powered oral hygiene devices over the gold standard dental floss and manual toothbrush. Improved oral hygiene parameters after 1 year may indicate implementation of newly acquired oral-hygiene skills during the 4-week instruction phase.

## Introduction

90% of the global population suffers from gingivitis [1–3]. Despite the availability of a broad range of oral hygiene products and increasing oral health and hygiene competency [3], the prevalence of gingivitis prevalence [2]. A study observing toothbrushing efficacy in adolescents concluded that removal of plaque was poor despite the high frequency of daily toothbrushing, even though participants were asked to clean their teeth to the best of their ability [4]. Reasons for this were poor brushing methods and a lack of motivation, knowledge, or ability to carry out efficient brushing movements [4, 5]. Recommendations regarding oral hygiene products and behavior consist of toothbrushing with a manual or powered toothbrush twice a day, with an even distribution of brushing time across all reachable surfaces [4, 6], as well as an efficient daily usage of interdental brushes or dental floss [1]. Flossing in addition to toothbrushing can lead to a reduction in gingivitis [7]. In cases where interdental flossing is not a realistic measure, other interdental cleaning devices may be useful in addition to the daily bushing routine. Interdental brushes are first choice if interdental tissues in narrow interdental spaces will not be damaged [1]. Alternatively, different water flossers and the more recent microdroplet devices (such as Philips AirFloss Pro®) are available, which aim to be more comfortable to use for interdental cleaning. Several authors have stated a reduction in gingival inflammation after using microdroplet devices [8–10].

Behavioral changes in daily oral hygiene routines is a first step to overcome these oral hygiene deficits. It is well known that many people find it difficult to adjust to recommended daily oral hygiene routines, and long-term adherence to these recommendations deteriorates quickly [7, 11, 12]. This difficulty particularly occurs when products are too complicated to use correctly [12] or recommendations are not made based on patients’ individual preferences [13]. Additionally, a lower socioeconomic status often limits the possibility of affording high-priced oral hygiene products and can correlate with a lack of knowledge regarding oral hygiene products [3].

To understand behavioral changes in patients, several definitions have been described. Compliance is defined by Cramer et al. as “the extent to which a patient acts in accordance with the prescribed interval and dose of a dosing regimen” [14]. Adherence is a less stringent term that may be used instead of compliance. Compliance is often documented as good or poor, mainly using percentages, where 80% is the cut-off point [14, 15]. In contrast, concordance describes a cooperative relationship between doctors and patients, to reach a set health goal together. In this way, patient preferences, fears, and concerns about a treatment choice are important [16, 17].

Most diseases of the oral cavity are preventable, at least in their severity, but only if preventive measures are routinely implemented [18]. In the context of daily oral hygiene recommendations, continuous concordance to measures such as toothbrushing or interdental care is an important success factor regarding lifelong oral and dental health. We know that 30–65% of health information provided by medical professionals will be forgotten within one hour after the appointment [11, 12]. For some medical conditions, non-adherence averages up to 50% [19]. Since adherence with daily brushing and interproximal care is the most essential factor for stable oral health, more effort must be made to investigate how concordance between patients and professionals can be achieved, and whether improved concordance leads to improved oral hygiene measures [1].

Convenient oral hygiene products, such as powered toothbrushes or a microdroplet device, show at least similar efficacy compared to use of a manual toothbrush or flossing with dental floss [1, 20]. However, both interdental care and use of a manual or powered toothbrush are technique-sensitive procedures [21–23]. Evaluation of patient acceptance of short-term use found that a microdroplet device was superior to dental floss [24]. Understanding patient preferences in their daily oral hygiene routine is important to provide advice when selecting oral hygiene products. However, less is known about patient acceptance and efficacy of a combination of powered oral hygiene devices for interdental care and brushing. It is important to understand whether patients with poor oral hygiene would achieve concordance and improve oral hygiene parameters while using powered oral homecare products in the long term.

Thus, the purpose of our study was to evaluate the long-term concordance with and acceptance of powered devices for oral homecare, as well as gingival health in gingivitis patients using powered devices in their everyday oral hygiene routine. We hypothesized that an easy-to-perform oral homecare-routine supported by powered oral hygiene devices would result in long-term concordance with use and an improvement in clinical oral hygiene parameters in gingivitis patients.

## Materials and methods

### Study design and methodology

**Fig. 1:**
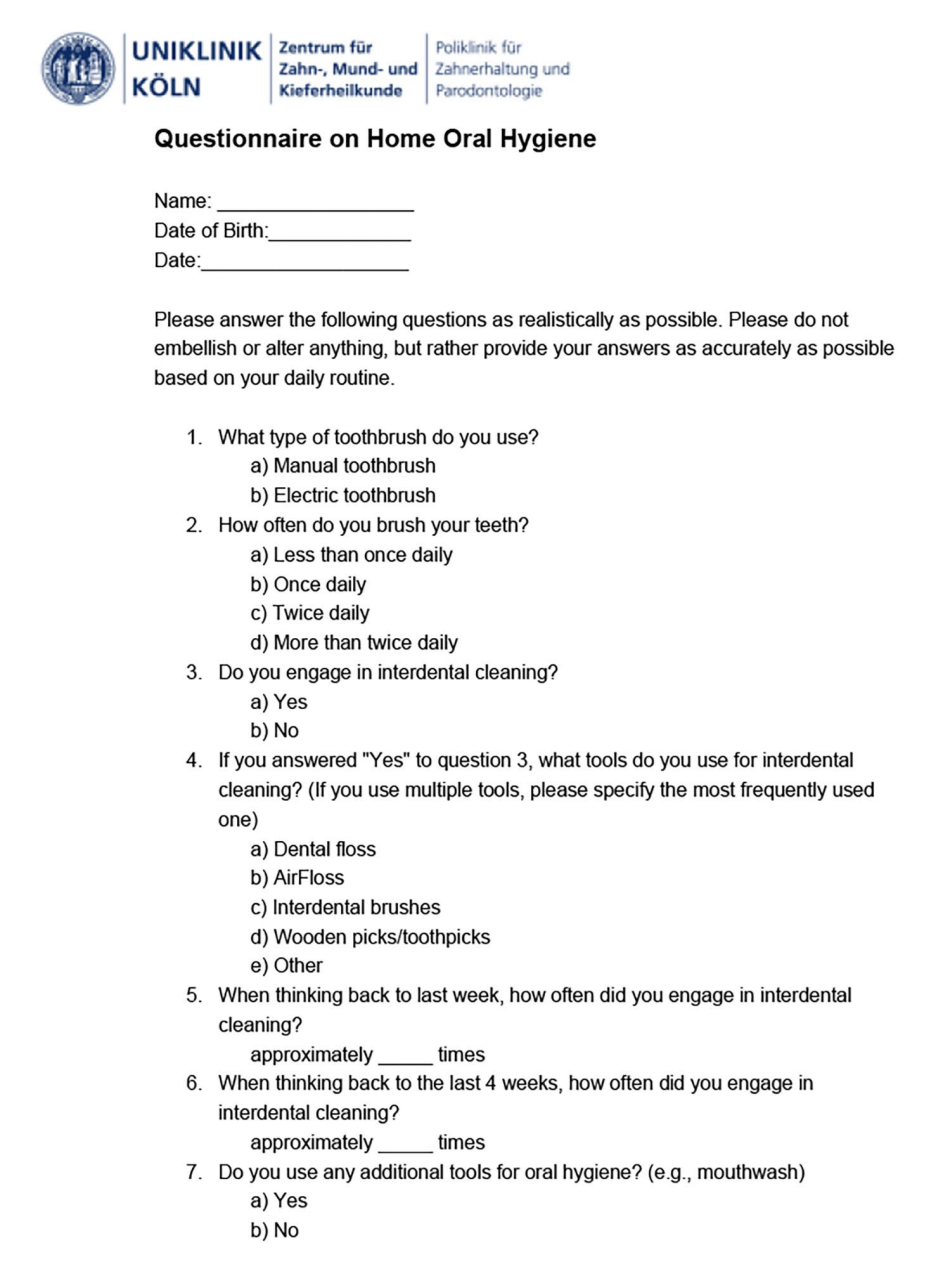
Questionnaire developed based on questions regularly asked regarding the patient’s oral hygiene routine during check-up appointments at the Polyclinic of Operative Dentistry and Periodontology, University of Cologne, Germany

We carried out a prospective, observational study divided into two phases. Prior to the study, all participants underwent routine dental assessment where oral hygiene indices were evaluated. The study began with a motivational trial (MT) phase, where efficacy, safety, and short-term acceptance were evaluated. In this phase, participants were randomly assigned to three groups (group 1: Sonicare® powered toothbrush & AirFloss Pro® (both Philips Nederland B.V., Netherlands) filled with water; group 2: Sonicare powered toothbrush & AirFloss Pro filled with Listerine® mouth rinse; group 3: manual toothbrush & dental floss). For 4 weeks, participants used the oral hygiene combination to which they were assigned (Fig. 1). The MT phase was designed to evaluate whether combinations of powered devices were at least as efficient as the combination of dental floss and a manual toothbrush, as previously described by Stauff et al. [24].

AirFloss Pro is a microdroplet device designed to clean narrow proximal spaces. The integrated water tank has a capacity of 14 ml. Depending on the operation modus used, one, two, or three puffs with 110 µl can be shot through a proximal space, using a nozzle to correctly locate the proximal space. AirFloss Pro should be used at least once a day for effective proximal hygiene [19]. The Sonicare Philips FlexCare toothbrush with the “ProResults C1” brush head is a powered toothbrush. To clean teeth and gingiva effectively, it should be used at least twice a day for 2 min, based on current literature [25]. All participants were instructed to use the toothbrush in “clean” mode [22]. Waxed dental floss (OralB Essential floss waxed, Procter & Gamble Service GmbH, Germany) was used as gold standard in interdental cleaning devices. The medium-hard toothbrush used (Friscodent M + C Schiffer GmbH, Germany) is available for purchase at a German supermarket and thus is an often-purchased product.

Assigned products were demonstrated with instructions for use by trained staff at the baseline of the MT phase, to enable effective use and minimize any potential danger of self-harm in all oral areas (incisive, premolars, molars). All members of staff were members of the postgraduate periodontology program at the Polyclinic of Operative Dentistry and Periodontology, University of Cologne, Germany. The control group also received the same demonstration and training on use of the powered devices during reevaluation of the MT phase (reevaluation 1). Therefore, all participants were instructed on the potential use of a combination of powered devices before the observational trial (OT) phase began.

At the reevaluation of the MT phase, all participants received the sonic toothbrush and microdroplet device and the unsupervised OT phase began. Participants were able to use their oral hygiene products of choice for 1 year. No specifications were made regarding the type or combination of products. At baseline, none of the participants had stated that they used any additional oral hygiene products (especially mouth rinse); therefore, any potential bias due to their ability to reduce plaque was minimized. After 1 year of unsupervised use, long-term clinical outcomes and concordance were investigated during a routine dental appointment by examination of oral hygiene indices and questionnaires (reevaluation 2). All oral examinations were performed at the Polyclinic of Operative Dentistry and Periodontology, University of Cologne, Germany.

The primary outcome was long-term concordance. Concordance and acceptance were evaluated using questionnaires about the patients’ oral hygiene routine, which were completed by participants prior to the MT phase (baseline), after the MT phase (reevaluation 1), and at the end of OT phase (reevaluation 2). Questions were asked regarding dental and interdental cleaning habits based on frequently asked questions regarding patients’ oral hygiene routine at check-up appointments at the Polyclinic of Operative Dentistry and Periodontology, University of Cologne (i.e., “Which type of toothbrush do you use?” or “Do you engage in interdental cleaning?”) (Fig. 1). Secondary outcomes were acceptance, the Rustogi-modified Navy Plaque Index (RMNPI) [26], the Quigley-Hein Index (QHI) [27, 28], and the papillary bleeding index (PBI) [26–29].

The clinical trial was approved by the local ethics review board of the University of Cologne, Germany (study number: 17–206) and registered (09.04.2021, DRKS00011619). The study design was in accordance with the Declaration of Helsinki (2001) and was carried out following Good Clinical Practice Guidelines (ICH-GCP). All participants gave written consent prior to baseline appointments after being informed about the contents, aims, and duration of the trial.

### Study population

Screening for participants used an announcement poster in the dental clinic of the University Hospital of Cologne, Germany, over a duration of 6 weeks. Participants meeting the following criteria [24] were included: (i) self-reported irregular use of interdental hygiene products (questionnaire data regarding regular oral hygiene routine and QHI > 0); (ii) PBI ≥1); (iii) no caries lesions (International Caries Detection and Assessment System (ICDAS) > I_2_) or restauration margins proximal to the first premolar (second if the first premolar was removed); (iv) no interdental clinical attachment loss and narrow interdental spaces; (v) interest in participation and written consent. Exclusion criteria were defined as: (i) periodontal disease (Community Periodontal Index (CPI) ≥ 3) or health (CPI 0); (ii) regular use of antiseptic mouth rinses; (iii) smoker (≥10 cigarettes/day); (iv) consumption of medication known to affect gingival health (antibiotic, calcium channel blocker, immunosuppressive) in the 3 months prior to the study; (v) dental professionals.

### Randomization and allocation concealment

Randomization of all participants into three groups during the MT phase was carried out by the senior investigator (S.H.M.D.), using a random, computer-generated list in sealed envelopes (Sealed Envelope LTD. 2018, available from https://www.sealedenvelope.com). The calibrated examiners were blinded regarding group allocation and the oral hygiene products used. A member of staff, who was not involved in clinical examinations during the study, carried out the randomization so that allocation concealment could be achieved.

### Adherence and patient acceptance after 4 weeks

At baseline, all participants were asked to complete a questionnaire regarding their usual daily oral routine. Questions were set based on a questionnaire used in a previous study at our clinic [24]. To evaluate adherence, participants were asked to keep an oral hygiene diary during the 4-week duration of the MT phase. All diaries were collected at the first recall appointment and checked for completion. After the MT phase, participants were asked to complete a questionnaire regarding self-reported efficacy and acceptance of their assigned oral hygiene cleaning routine. Questions such as “How do you perceive the usage of AirFloss Pro®/dental floss?” (with multiple choice answers) or “Do you wish to continue the usage of your assigned proximal cleaning devices?” were answered by participants. These questions were set based on a questionnaire used in a previous study at our clinic [24].

### Concordance with oral hygiene products and routine after 1 year

Concordance with the oral hygiene routine was evaluated using questionnaires at patient appointments 1 year after baseline (reevaluation 2). Patients were asked to name the type of oral hygiene products used (type of toothbrush and interdental care), as well as frequency of usage (daily and/or weekly). Items of the questionnaire were based on questions regularly asked about the patient’s oral hygiene routine during check-up appointments at the Polyclinic of Operative Dentistry and Periodontology, University of Cologne. Examples of questions asked include “Do you clean your interdental spaces?” or “Which type of interdental cleaning device do you prefer?”. Similar questions were asked regarding toothbrushing routines (Fig. 1).

### Clinical parameters

Oral health indices such as the PBI, RMNPI, and QHI and safety were elevated and documented by I.S./D.D. in a case report form. All investigators were members of the postgraduate periodontology program and trained by the senior investigator S.H.M.D. in the section of periodontology. The PBI was documented buccal mesial and distal of the tested premolar teeth using a periodontal probe (PCPUNC15, Hu-Friedy, Mfg.Co., LLC, Tuttlingen, Germany) [29]. Dental plaque was visualized mesial and distal of the tested premolar tooth, using a plaque elevator solution (Mira-2-Ton, Miradent, Hager & Werken GmbH & Co. KG, Germany). Biofilm was evaluated using the QHI and RMNPI in the proximal and gingival areas A/D and F/C. The amount of plaque at each area was documented photographically and in writing [26–28].

### Safety

Routinely in all clinical studies, safety protocols are mandatory to assess and document potential study induced harms. In this case, the expected unwanted side effects of using oral home care devices were gingival lesions, i.e., gingival abrasion [30–35]. A case report form was designed to document these lesions if they occurred. These were documented at all oral examination appointments and characterized by localization and extent.

### Sample size

Sample size calculation was based on the MT phase. Previous studies regarding changes in plaque indices over time showed an effect size of Cohen’s d = 1.41 [36]. Assuming an effect size of 1.0, a power of 95%, and a beta error of 5% when comparing baseline values to measurements after 4 weeks, a sample size of 16 was estimated [37]. This sample size was needed to show that the chosen oral hygiene products were safe and effective to use. During the OT phase, high numbers of dropouts were expected due to the long-term appointments after 1 year. Therefore, subjects were initially recruited over a duration of 6 weeks, resulting in 31 participants.

### Statistical analysis

Statistical analysis was carried out at participant level (unit of analysis), using SPSS statistics 27.0 software (SPSS Inc., Chicago, IL, USA). Statistical significance was indicated when *p* < .05 was reached.

Concordance of patients with their daily oral hygiene routine was derived from completed questionnaires and listed in descriptive tables. For all three groups (MT phase), mean values (standard deviations, SD) for PBI, QHI, and RMNPI were calculated. Differences between groups at baseline and recall appointments were investigated using a one-way ANOVA test. Within-group variations of the parameters between baseline, first, and/or second recall appointments were analyzed using Wilcoxon signed rank test. Missing values were processed using the last-observation-carried-forward principle.

## Results

**Fig. 2:**
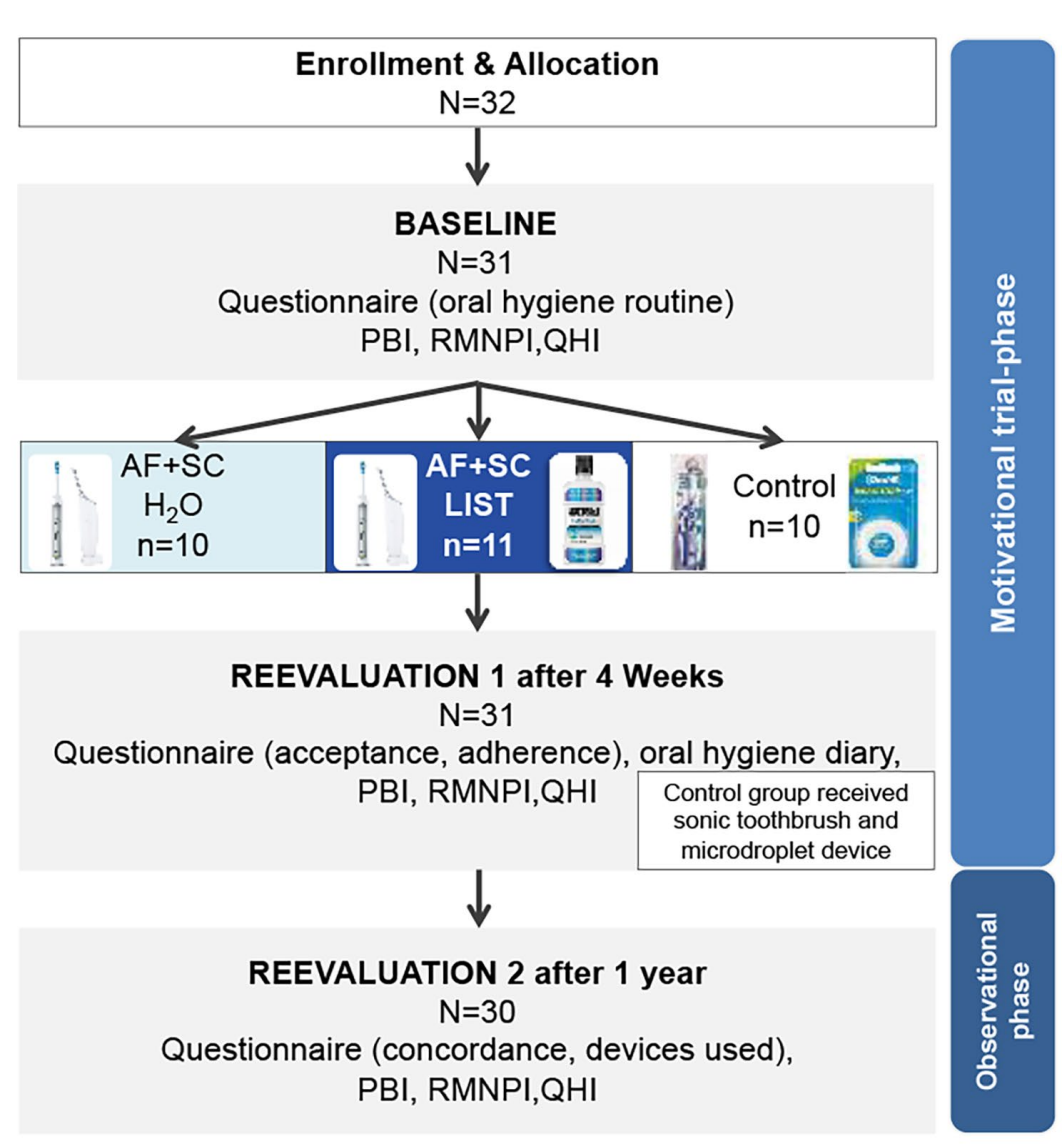
Study flow-chart demonstrating the duration and different phases of the investigation

All 31 participants included in the study finished the MT Phase (Table 1). Twenty-seven of these (52% female, mean age 33 (SD 14) years) finished the OT phase (period of recruitment prior to MT phase: 12 April 2021 to 23 May 2021) (Fig. 2). Four participants did not attend the dental appointment after 1 year (pregnancy *n* = 2, relocation *n* = 2). Three of these patients returned the questionnaire (via email) regarding their daily oral hygiene routine.

**Table 1:** Characteristics of participants at baseline

	Total (*N* = 31)	Microdroplet device & PTB (Listerine®) (H_2_O)	Microdroplet device & PTB (Listerine®)	Control (Dental floss & MTB)	*p*-value*
Age					0.372
Mean ± SD	32 ± 14	29 ± 7	30 ± 13	37 ± 19	
Range	19–82	20–45	19–60	21–82	
sex, *n* (%)					
male	13 (42)	5 (50)	2 (18.2)	6 (60)	
female	18 (58)	5 (50)	9 (81.8)	4 (40)	
DMFT					0.929
Mean ± SD	8.9 ± 7.8	8.3 ± 8.4	9.6 ± 7.6	8.8 ± 8.4	
Range	0–22	0–22	0–21	0–21	
PBI					0.999
Mean ± SD	1.6 ± 0.5	1.6 ± 0.5	1.6 ± 0.5	1.6 ± 0.6	
Range	1–3	1–3	1–3	1–3	
RMNPI					
Mean ± SD	1 ± 0	1 ± 0	1 ± 0	1 ± 0	
Range	1–1				
QHI					0.162
Mean ± SD	2.3 ± 1.1	2.0 ± 0.7	2.8 ± 1.5	2.1 ± 0.6	
Range	1–5				

**Fig. 3:**
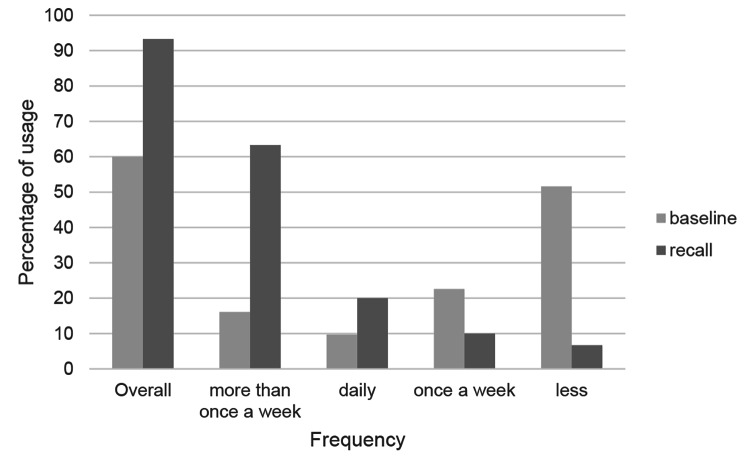
Frequency of usage of interdental cleaning devices after the 1-year OT phase compared to baseline

*p < .05, analyzed with ANOVA. DMFT, decayed, missing, filled teeth; MTB, manual toothbrush; PBI, papillary bleeding index; PTB, powered toothbrush; QHI, Quigley-Hein index; RMNPI, Rustogi modified Navy Plaque index; SD, standard deviation;

At baseline, 74.2% of participants stated they used interdental cleaning devices less than once a week (Fig. 3). The main reason reported (38.7%) was “too hard to use”.

### Adherence and patient acceptance after 4 weeks

After the MT phase, all participants in the AirFloss Pro groups used the microdroplet device daily (control group: dental floss 70.0%) and said they would continue to use it after finishing the MT phase (control group: dental floss 80.0%). Overall, 85.7% of patients in the AirFloss Pro groups said they had a “comfortable” feeling while using AirFloss Pro (control group: dental floss 20.0%) (Table 2).

**Table 2:** Self-reported acceptance and adherence of participants after the MT phase at 4 weeks, frequency of usage, and willingness to continue usage of their assigned interdental cleaning device

	AirFloss Pro® (H2O) *n* (%)	AirFloss Pro® (Listerine®) *n* (%)	dental floss *n* (%)
Daily usage	10 (100.0)	10 (90.9)	7 (70.0)
Willingness to Use after MT-Phase	9 (90.0)	11 (100.0)	8 (80.0)
effective	7 (70.0)	10 (90.9)	9 (90.0)
feel efficacy	8 (80.0)	9 (81.8)	7 (70.0)
see efficacy	2 (20.0)	7 (63.6)	6 (60.0)
less blood	7 (70.0)	10 (90.9)	4 (40.0)
no feelable difference	2 (20.0)	0 (0.0)	2 (20.0)
no difference at all	4 (40.0)	0 (0.0)	4 (40.0)
comfortable	8 (80.0)	10 (90.9)	2 (20.0)

At baseline, 41.9% of participants used a powered toothbrush. After the MT phase, 61.9% of participants in the AirFloss Pro groups thought the experience of using a powered toothbrush was “very comfortable” and 95.2% would continue brushing with a powered toothbrush after the MT phase.

### Concordance with oral hygiene products after 1 year (primary outcome)

During their annual check-up after 1 year, 93.3% of participants reported that they performed interdental cleaning on a regular basis (compared to 60.0% at baseline), and 63.3% stated that they cleaned their interdental spaces more than once a week (Table 3).

**Table 3:** Preferred interdental cleaning devices after the 1-year OT phase, including the frequency of usage during the last 7 days and 4 weeks (*n* = 27)

interdental	*n* (%)	Frequency last week	frequency last 4 weeks
AirFloss Pro	16 (53.3)	3.6	14.4
dental floss	9 (30.0)	3.8	14.8
Interdental brush	3 (10.0)	5.0	20.7
none	2 (6.7)	—	—

After 1 year, 53.3% of participants preferred AirFloss Pro and 30.0% used dental floss for their daily oral hygiene (Table 3). Frequency of usage of AirFloss Pro and dental floss was almost similar. The main reason for using AirFloss Pro was a “clean feeling” (33.3%). Reasons against using AirFloss Pro included “other products more effective” (23.3%) and other reasons (23.3%; for example, “nozzle location of device too complicated”, “changing habits in oral hygiene not possible”).

The percentage of patients using a powered toothbrush increased from 41.9% at baseline to 90.0% after 1 year. Frequency of usage twice daily increased from 71.0% at baseline to 80.0% at 1 year (Table 4).

**Table 4:** Toothbrushes used and frequency of usage at the baseline appointment and the individual patient recall after the 1-year OT phase (reevaluation 2)

	Baseline, *n* (%)	Recall, *n* (%)
Powered Toothbrush	13 (41.9)	27 (90.0)
Manual toothbrush	18 (58.1)	3 (10.0)
Usage once a day	9 (29.0)	6 (20.0)
Usage twice a day	22 (71.0)	24 (80.0)

### Oral hygiene parameters

After the MT phase (including all patients), the PBI (*p* = .000), QHI (*p* = .000), and RMNPI (*p* = .003) were significantly decreased compared to baseline. Both AirFloss Pro groups (microdroplet device/powered toothbrush) and the control group (dental floss/manual toothbrush) showed a significant decrease in PBI after demonstration and 4 weeks of using their assigned products (AF + SC (H2O) *p* = .004; AF + SC (List) *p* = .003; control *p* = .004). Regarding RMNPI, only the AirFloss Pro groups showed significant better results (AF + SC (H_2_O) *p* = .034; AF + SC (List) *p* = .034) compared to participants using dental floss and a manual toothbrush (*p* = .317). Similar results were observed when measuring QHI after the MT phase (AF + SC (H2O) *p* = .020; AF + SC (List) *p* = .011; control *p* = .960).

**Fig. 4:**
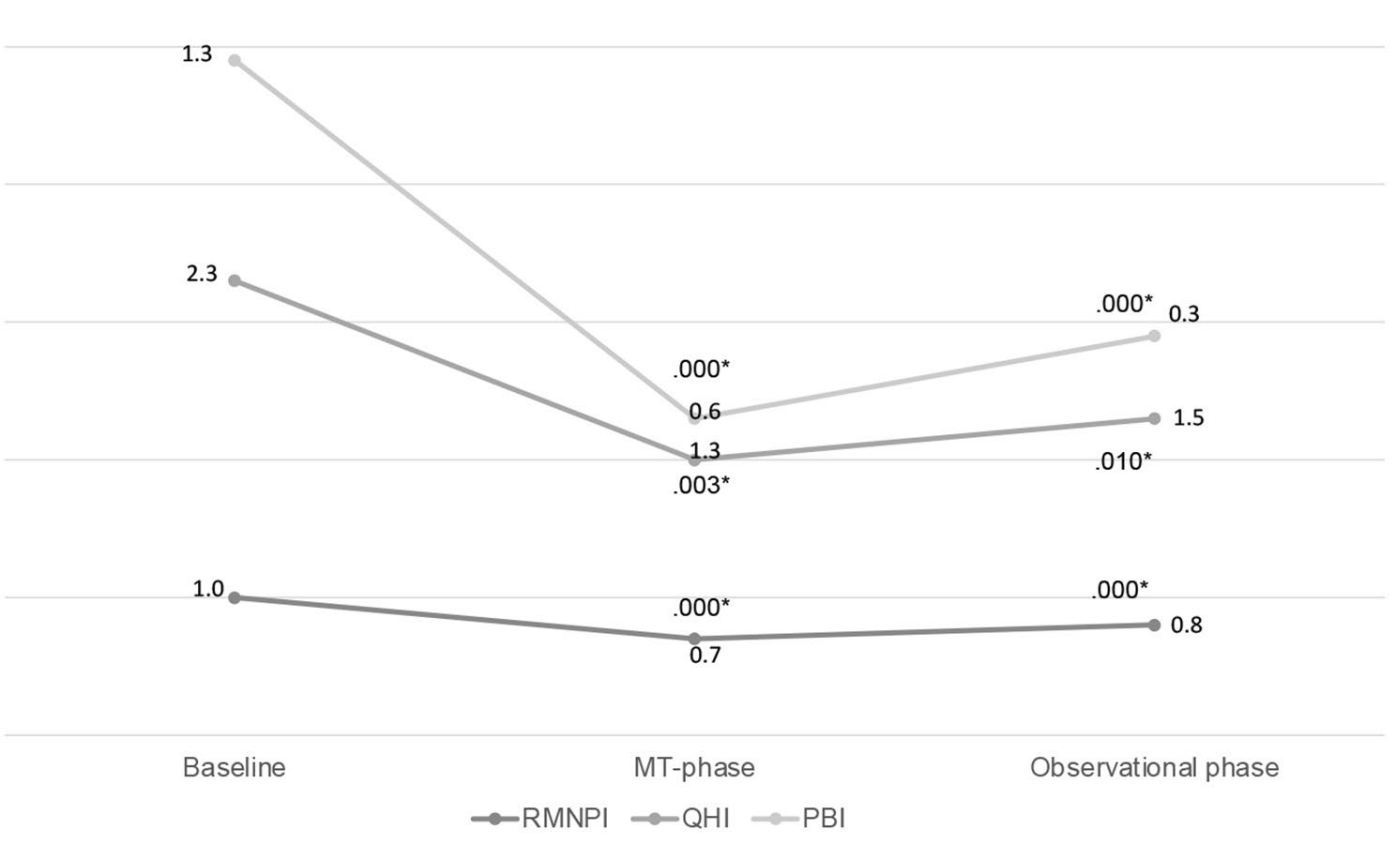
Papillary bleeding index (PBI), Rustogi Modified Navy Plaque Index (RMNPI), and Quigley-Hein Index (QHI) at baseline, after the MT phase at 4 weeks (reevaluation 1), and the OT phase after 1 year (reevaluation 2). ^*^p < 0,05; analyzed with ANOVA

After 1year, the improvement in oral hygiene parameters remained in the 27 patients (PBI: *p* = .000; RMNPI *p* = .010; QHI *p* = .000) (Fig. 4).

### Safety

No gingival injuries or abrasions were observed at any appointment.

## Discussion

The purpose of our study was to evaluate long-term concordance with and acceptance of unsupervised use of powered devices for oral homecare, and the impact on gingival health in patients with papillary bleeding using powered devices in their everyday oral hygiene routine. Our results suggest that oral hygiene indices remained improved over a period of 1 year after providing powered oral homecare devices and oral hygiene training.

The randomized MT phase was scheduled for 4 weeks. As shown in previous studies, a duration of 4 weeks was a suitable period to evaluate short-term changes in patient motivation, as well as clinical bleeding indices and biofilm accumulation [38, 39]. According to the guidelines of the American Dental Association, 4 weeks is long enough to evaluate the efficacy of oral hygiene devices such as a microdroplet device and to observe changes in gingival health [40, 41]. The OT phase took place over an approximate duration of 1 year. We chose the participants’ individual recall appointment to evaluate their actual daily oral routine with minimized disruptive influences such as the Hawthorne effect. In addition, it has been stated that instructions regarding oral hygiene routines could change patient behavior for up to 3 months [32]. As several previous studies lasted over 6 months, we doubled the duration to 1 year to mirror patients’ oral homecare routine as precisely as possible [42].

In our investigation, patient acceptance of a microdroplet device was high after 4 weeks, and all participants in AirFloss Pro groups stated continuous usage after the MT phase. These results reflect a previous evaluation of the use of a microdroplet device for 4 weeks [24]. In our study after 1 year, 93.3% of participants cleaned their interdental spaces and 53.3% used AirFloss Pro. These findings are supported by other studies, where patients rated the use of AirFloss Pro in daily routine as a positive adjunctive in the short-term and after 6 months [24, 42]. Our results are also supported by a recent mixed methods study, where patients in focus group discussions reported a lack of motivation or knowledge of usage regarding interdental care products such as dental floss; patients recommended improvement of interdental devices such as floss or interdental soft picks to make the product easier to use and more convenient [43]. In our study, participants perceived greater comfort when using AirFloss Pro filled with Listerine mouth rinse, which may generate an even cleaner feeling that may be caused by the fresh taste. It needs to be considered that Listerine mouth rinse may lead to plaque reduction and reduction of gingival bleeding and thus a reduction of gingivitis [44]. However our results did not reflect such alterations as participants who used AirFloss Pro filled with Listerine mouth rinse did not show significantly different changes in oral hygiene indices. 

After 4 weeks and 1 year, participants showed significantly reduced bleeding and plaque indices. Equivalent results have also been shown in literature, where microdroplet devices were able to reduce gingival bleeding after 4 weeks and 6 months [20, 42]. This may be attributed to improved oral homecare, especially regular interdental cleaning routine with the microdroplet device. The use of the microdroplet devices may cause an alteration in composition of dental plaque, a reduction of biofilm thickness after usage, alteration in the hosts’ immune response, or stimulation of the gingiva [9]. Furthermore, some participants may have used chemical plaque control in addition to their oral homecare routine, which is also able to reduce dental biofilm and therefore gingivitis [44]. All these mechanisms support the transition of an incipient dysbiosis to a healthy symbiosis [45].

Overall, 90.0% of our participants reported using a powered toothbrush after 1 year. Several other authors have stated that powered toothbrushes are superior to brushing with a manual toothbrush with respect to reduction of plaque and gingivitis [46, 47]. Additionally, a long-term comparison of three nationwide, cross-sectional surveys over 17 years showed more caries-free teeth surfaces and more remaining teeth in patients who used a powered toothbrush and interdental care [48]. Another long-term observation found a correlation between use of a powered toothbrush and reduction in pocket depths and less progression in clinical attachment loss after 11 years [49].

However, none of the previous long-term observations have focused on the impact of either the toothbrush or the interdental cleaning aid used in terms of cleaning efficacy. This raises the question whether use of a powered toothbrush or a microdroplet device alone would result in reduced bleeding and plaque indices. Possible answers may be found regarding the different types of plaque indices evaluated. The QHI focuses on the entire buccal site of the tooth, representing the ability to reduce plaque by a toothbrush [28]. The RMNPI areas A/D and F/C represent the interproximal marginal gingival space of a tooth, therefore mirroring the efficacy of proximal cleaning actions [26]. Both indices were significantly reduced after 4 weeks, but only in the AirFloss Pro groups using a powered toothbrush and microdroplet device; this indicates sufficient plaque control of both powered devices in their specific areas of the tooth. It should be noted that the ability of dental floss to clean proximal spaces of premolars, especially in the approximal retraction, is reduced due to their anatomical design, even though interdental spaces were narrow.

After 1 year, at the participants` individual dental check-up, concordance with powered devices was high. The percentage of participants cleaning their interdental spaces increased to 93.3% (baseline 60.0%), with 53.3% of patients preferring to use the microdroplet device (30.0% dental floss). 20% of participants even stated that they cleaned their interdental areas daily. One possible explanation might be implementation of adequate brushing and interdental cleaning skills into the patients’ everyday dental cleaning routine. As shown before, (repeated) professional dental instructions can lead to an increased understanding and use of the methods instructed [50, 51]. Planning actions, such as planning when, where, and how to use the dental hygiene method of choice, can result in increased patient adherence [52]. It should be noted that in some previous studies, adherence was defined only as the daily use of dental floss in contrast to the definition of concordance [52, 53]. Our results may indicate a long-term behavioral change, one of the highest goals in medical treatment but especially in dentistry because biofilm control is a main risk factor for most oral diseases and can be reduced by an adequate oral homecare routine.

Our investigation has some limitations. The questionnaires evaluating concordance at reevaluation 2 were self-designed, based on questions regularly asked at dental appointments in our clinic. Tisnado et al. evaluated the concordance between medical records and patients’ self-reports to multiple medical items [54]. They found a high concordance and patients were able to report with good sensitivity. In contrast to this study, our participants chose their own preferred combination for oral homecare. It might be expected that reporting of a preferred product combination was high, even though the questionnaires were not validated. A supervised, individual, patient-centered, 4-week motivational phase is hard to implement in everyday dental care because it is time consuming and ties up human resources. This highlights the need for adjustment regarding prevention concepts in dental settings. For example, professionally teaching of oral homecare may be a valuable addition during regular dental check-ups. Another limitation is that our results are not applicable to patients suffering from periodontal disease and therefore loss of papillae and open interdental spaces [1]. Patients who smoked fewer than ten cigarettes per day were eligible to participate in the study and were not distributed equally. Tobacco smoke reduces microvascular vasoconstriction and causes fibrosis of the gums through systemic circulation of components of cigarette smoke, as well as local uptake. Such consequences may mask gingivitis indices in the short and long term [55]. Furthermore, the investigation was carried out with dental floss as control. A local (German) guideline focusses on at-home mechanical biofilm management in the prevention and therapy of gingivitis [56]. Even in patients without clinical attachment loss, interdental brushes are more effective at biofilm reduction than dental floss. Dental floss should only be considered if narrow interdental spaces are present. In future studies, interdental brushes will serve as control of choice. Moreover, other areas are harder to reach during oral homecare (such as areas with orthodontic retainers or molars), which might make our results less applicable. As stated in recent literature, patient-reported outcomes such as oral wellbeing or willingness-to-pay need to be taken into account when investigating the treatments of oral diseases [57]. Until now, most short- and long-term investigations regarding oral hygiene measurements focus on clinical outcomes; recently, patient preferences have been gaining more attention in this area [57]. Actual changes in the daily routine of patients for prevention of oral diseases can only take place if barriers to achieve these goals are low or prevention measures are elaborated In our investigation, we showed how patient behavior can change when providing them with powered, convenient oral healthcare products such as a powered toothbrush and a microdroplet device after professional instruction. Prevention of an illness or treatment at an early stage is less expensive than treating the actual illness [2]. In particular, treatment of gum diseases such as gingivitis using adequate daily proximal care can prevent the prevalence of periodontitis [1]. Future investigations should be carried out on a wider scope, exploring how the combination of professional advice for dental homecare can be combined most efficiently with oral healthcare products.

## Conclusion

In this study, an initial 4-week motivational trial phase, which included oral hygiene instructions and individual support, led to improved interdental cleaning and brushing skills and implementation of newly acquired habits in the mindset of patients. In the long term, if patients had a free choice of different devices offered, patients with initial gingival bleeding preferred the unsupervised use of powered oral hygiene products over manual devices, including dental floss. This choice resulted in improved oral hygiene indices after 1 year.

## Data Availability

The datasets used and analysed during the current study are available from the corresponding author on reasonable request.
